# Brain structure–function decoupling and neuromuscular coordination during gait and sit-to-stand in healthy aging

**DOI:** 10.3389/fnagi.2026.1932354

**Published:** 2026-10-20

**Authors:** Qiaowu Xiong, Weixing Zhong, Xuan Chen, Min Yang, Yuanxun Lin, Hua Lu, Qinhao Fang, Zhaoxing Liu, Lei Qian, Yikai Li, Weidong Cheng

**Affiliations:** 1 School of Traditional Chinese Medicine, Southern Medical University, Guangzhou, China; 2 School of Basic Medical Sciences, Southern Medical University, Guangzhou, China

**Keywords:** aging, basal ganglia, gait, intermuscular coherence, mobility decline, muscle synergy, sit-to-stand, structure–function decoupling

## Abstract

**Background:**

Age-related decline in lower-limb motor function reflects complex interactions between central brain networks and peripheral neuromuscular coordination. This study examined how structural–functional brain organization relates to muscle coordination, intermuscular coupling, and biomechanical control during gait and sit-to-stand (STS) tasks.

**Methods:**

In this exploratory multimodal study, thirteen healthy young adults and sixteen healthy older adults underwent resting-state fMRI, DTI, surface electromyography, motion capture, plantar pressure, and ground reaction force measurements. Muscle synergy similarity, intermuscular coherence (IMC), IMC network efficiency, center-of-pressure (COP), ground reaction force (GRF), and structure–function decoupling index (SDI) were quantified and analyzed.

**Results:**

Older adults exhibited lower muscle synergy similarity and reduced gait-related IMC and IMC network efficiency, together with altered COP and GRF patterns. Regional SDI alterations were concentrated in basal ganglia/striatal, cingulate, and insular regions. In age-group-adjusted exploratory analyses, STS muscle synergy similarity and mean IMC showed nominal positive associations with SDI in the right ventromedial putamen and right dorsal area 23; however, these associations did not survive FDR correction across the candidate ROIs.

**Conclusion:**

Healthy aging is associated with task-dependent reorganization of lower-limb neuromuscular control and selective basal ganglia-striatal structure–function alterations. These findings provide multimodal evidence of parallel central and peripheral reorganization during healthy aging and identify candidate brain-muscle associations for future longitudinal and confirmatory investigation.

## Introduction

Mobility decline is one of the most clinically relevant manifestations of healthy aging. Even in the absence of overt neurological disease, older adults frequently show slower walking, impaired postural transitions, reduced lower-limb strength, decreased neuromuscular coordination, and poorer balance control, which together increase the risk of falls, disability, loss of independence, and reduced quality of life (Seidler et al., 2010; Rosso et al., 2013; Wang et al., 2024; Montero-Odasso et al., 2022; Rubenstein, 2006). Gait and sit-to-stand (STS) transitions are two representative daily motor behaviors: gait reflects rhythmic locomotor control and dynamic balance, whereas STS requires rapid weight transfer, antigravity force generation, and coordinated trunk-hip-knee-ankle control (Bohannon, 2012; Millor et al., 2013; Lord et al., 2013). Therefore, age-related alterations in these two tasks may provide complementary information about lower-limb motor decline during natural aging.

Traditionally, mobility decline in older adults has been attributed mainly to peripheral factors, such as sarcopenia, reduced muscle strength, joint degeneration, and impaired proprioceptive feedback (Cruz-Jentoft and Sayer, 2019; Larsson et al., 2019; Clark, 2015). However, accumulating evidence indicates that walking and postural control are not purely peripheral motor outputs but are regulated by distributed brain systems involving basal ganglia, sensorimotor cortex, cingulate cortex, insula, frontoparietal networks, cerebellum, and white matter pathways (Holtzer et al., 2014; Wilson et al., 2019; Yuan et al., 2015; Droby et al., 2022; Hsu et al., 2023). Structural and functional neuroimaging studies have shown that white matter integrity, resting-state functional connectivity, and brain network organization are associated with gait speed, gait variability, balance, and mobility decline in older adults (Yuan et al., 2015; Droby et al., 2022; Hsu et al., 2023; Alzaid et al., 2022; Tian et al., 2016). These findings suggest that age-related lower-limb motor decline should be conceptualized as a central–peripheral process rather than as an isolated musculoskeletal problem.

This central–peripheral perspective has important therapeutic implications. Interventions targeting the central nervous system, such as transcranial magnetic stimulation, transcranial direct current stimulation, and other non-invasive neuromodulation techniques, have been explored for improving gait and balance, while peripheral interventions, including resistance exercise, balance training, physical therapy, and sensorimotor rehabilitation, remain core strategies for preventing mobility decline and falls (Zhang et al., 2023; Tseng et al., 2024; Wei et al., 2025; Sherrington et al., 2019). Nevertheless, the selection of intervention targets remains insufficiently evidence-based. For central interventions, the optimal brain regions or networks to modulate are still unclear. For peripheral interventions, it remains uncertain which muscles, muscle groups, or neuromuscular coordination patterns should be prioritized. A key unresolved question is therefore how age-related changes in brain structure–function organization are linked to altered lower-limb neuromuscular coordination and biomechanical control.

Surface electromyography, motion capture, plantar pressure, and ground reaction force measurements can identify peripheral motor signatures of aging, including altered muscle coordination, impaired intermuscular coupling, abnormal center-of-pressure migration, and reduced force generation (Ting et al., 2015; Bizzi and Cheung, 2013; Magrath et al., 2022; Dos Santos et al., 2020; Dussault-Picard et al., 2024). Among these measures, muscle synergy similarity can quantify whether an individual’s muscle coordination pattern remains close to a young adult reference pattern, whereas intermuscular coherence can capture the degree of synchronized muscle–muscle coupling that may reflect shared neural drive (Ting et al., 2015; Bizzi and Cheung, 2013; Magrath et al., 2022; Dussault-Picard et al., 2024). These peripheral metrics are particularly relevant for identifying muscle-level and network-level targets for rehabilitation. However, they are rarely examined together with brain structure–function coupling metrics in the same participants.

Recent advances in multimodal neuroimaging provide an opportunity to bridge this gap. The structure–function decoupling index (SDI), derived from resting-state functional MRI signals constrained by DTI-based structural connectivity, quantifies the extent to which regional functional activity is coupled with or decoupled from the underlying structural connectome (Baum et al., 2020; Preti and Van De Ville, 2019). This measure may be useful for identifying brain regions where functional flexibility or structural constraint is altered with aging. If regional SDI is associated with peripheral neuromuscular coordination during gait or STS, it may help identify central nodes that are functionally relevant to lower-limb motor aging.

In this exploratory cross-sectional study, we recruited healthy young and older adults and acquired multimodal data, including resting-state fMRI, DTI, surface electromyography, motion capture, plantar pressure, and ground reaction force during barefoot gait and STS tasks. We examined: (1) whether healthy aging is associated with altered brain structure–function decoupling in motor-control-related networks; (2) whether older adults show reduced muscle synergy similarity, altered intermuscular coherence, and impaired COP/GRF control during gait and STS; and (3) whether central SDI alterations were associated with peripheral neuromuscular coordination metrics. Given the modest sample size and breadth of the multimodal measurements, the analyses were intended to generate hypotheses and identify candidate central-peripheral associations rather than confirm ROI-specific biomarkers or intervention targets.

## Materials and methods

### Participants

A total of 29 healthy participants were recruited for this cross-sectional observational study, including 13 young adults and 16 older adults. All participants were screened to ensure that they were able to complete MRI scanning, barefoot walking, STS testing, sEMG recording, plantar pressure/ground reaction force assessment, and motion capture procedures. Participants were excluded if they had a history of neurological disorders, lower-limb musculoskeletal diseases, previous lower-limb surgery, severe visual or vestibular dysfunction, contraindications to MRI scanning, or inability to walk independently.

The study protocol was approved by the Ethics Committee of Foshan Nanhai District People’s Hospital (No. 2023063), and all participants provided written informed consent before participation. The study was conducted in accordance with the Declaration of Helsinki. Demographic characteristics, including sex, age, height, weight, and body mass index (BMI), were recorded before the experiment.

### Sample-size considerations

The study was designed as an exploratory, hypothesis-generating multimodal investigation. The final sample comprised 29 participants who completed the full multimodal protocol and had complete, quality-controlled datasets across resting-state fMRI, diffusion MRI, surface electromyography, motion capture, plantar-pressure/ground-reaction-force, gait, and sit-to-stand assessments. No formal *a priori* sample-size calculation was performed. An analytical sensitivity analysis based on the noncentral t distribution was therefore used to characterize the statistical sensitivity of the available sample. Assuming a two-sided alpha of 0.05 and 80% power, the group sizes of 13 young and 16 older adults yielded an approximate minimum detectable standardized mean difference of Cohen’s d = 1.09 for a conventional two-group comparison. For an unadjusted bivariate correlation across all 29 participants, the corresponding minimum detectable effect was approximately |r| = 0.49. These estimates apply to nominal tests; covariate-adjusted models and multiplicity-corrected analyses would require larger effects for comparable power. Accordingly, ROI-level and cross-modal association analyses were considered exploratory and require confirmation in independent cohorts.

### Experimental protocol and movement tasks

The overall experimental design and multimodal data acquisition protocol are shown in Figure 1. Each participant completed the study protocol in a single visit. The experiment began with MRI acquisition, followed by biomechanical and neuromuscular assessments in the gait laboratory. Specifically, participants first underwent resting-state fMRI and diffusion tensor imaging (DTI) scanning (Figure 1A). After MRI acquisition, they were transferred to the gait analysis laboratory, where reflective motion-capture markers and surface electromyography (sEMG) electrodes were attached for synchronized motion, plantar pressure, ground reaction force, and muscle activity recording (Figure 1B).

**Figure 1:**
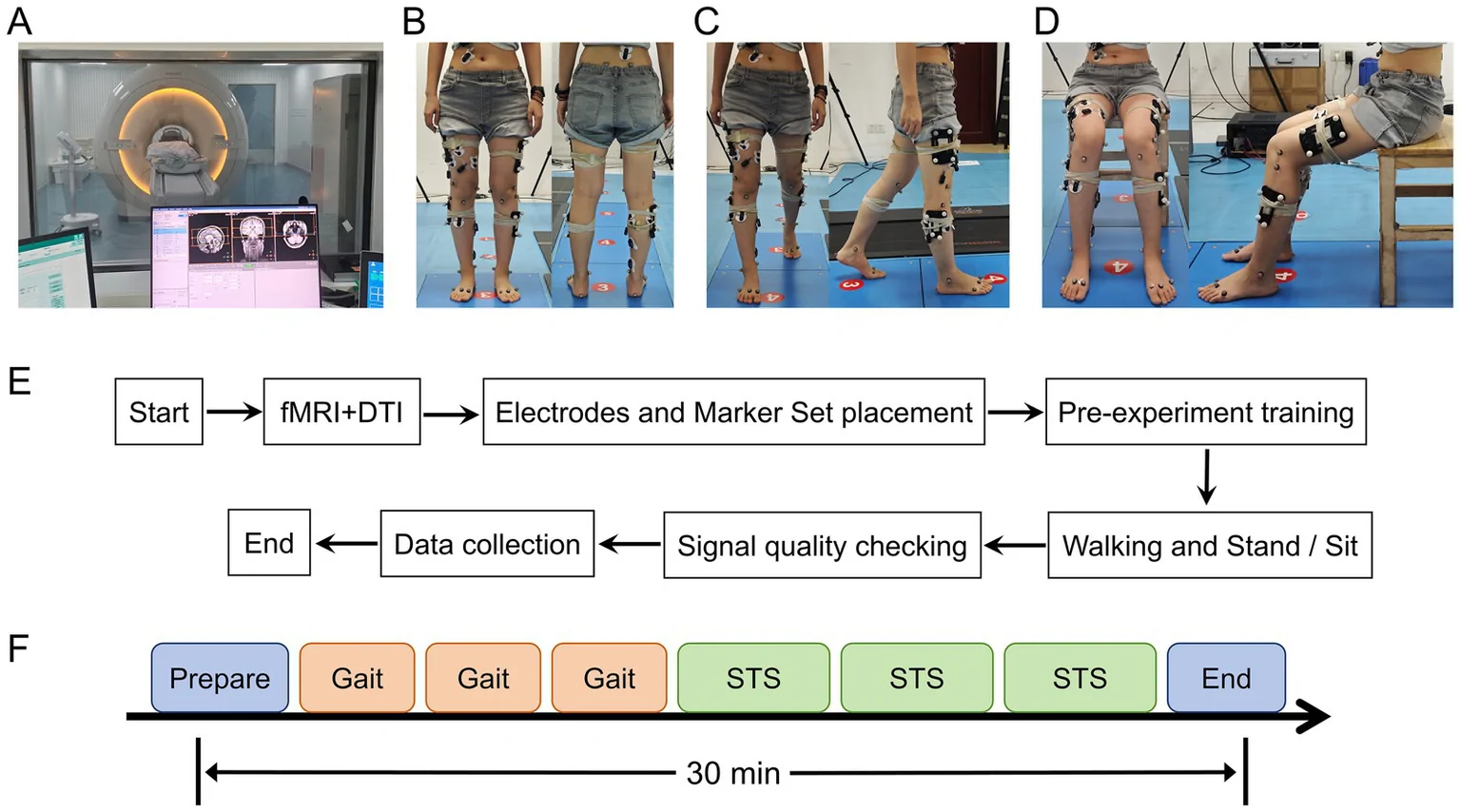
Experimental design and data acquisition protocol. **(A)** Representative MRI image acquisition. Resting-state fMRI and diffusion tensor imaging (DTI) were acquired before biomechanical testing. **(B)** Placement of surface electromyography electrodes and motion-capture markers before movement testing. Surface EMG sensors and reflective markers were attached according to the standardized lower-limb experimental protocol. **(C)** Representative scene of the barefoot walking task. Participants were instructed to walk naturally at a self-selected comfortable speed on the walkway. **(D)** Representative scene of the standing/sit-to-stand task. Participants performed repeated stand–sit–stand transitions on the plantar pressure/force platform. **(E)** Flow chart of the experiment within one visit. Participants first underwent fMRI and DTI scanning, followed by electrode and marker placement, pre-experiment training, walking and stand/sit tasks, signal quality checking, and synchronized data collection. **(F)** Timeline of the biomechanical experiment. After preparation, participants completed three walking trials and three sit-to-stand/stand-to-sit trials. The whole biomechanical testing session lasted approximately 30 min.

Surface EMG electrodes (2000 Hz, Mini Wave Infinity, Cometa, Italy) were attached after skin preparation, including shaving if necessary, gentle abrasion, and alcohol cleaning to reduce skin impedance. Electrodes were placed over the muscle bellies of 16 trunk and lower-limb muscles on the right side, with each electrode aligned along the direction of the muscle fibers. The recorded muscles included erector spinae (ES), external oblique (EO), gluteus maximus (GLM), rectus femoris (RF), vastus medialis (VM), vastus lateralis (VL), semitendinosus (SED), biceps femoris (BF), tibialis anterior (TA), fibularis longus (FL), extensor digitorum longus (EDL), gastrocnemius medialis (GM), gastrocnemius lateralis (GL), soleus (SOL), flexor hallucis longus (FHL), and abductor hallucis (AH) (Figure 2B). Reflective markers were placed according to a standardized lower-limb biomechanical protocol based on the CAST lower-limb model and the IOR foot model (100 Hz, Miqus M1, Qualisys, Sweden). This marker configuration enabled reconstruction of pelvis, hip, knee, ankle, and multi-segment foot motion, including the shank–calcaneus, calcaneus–midfoot, midfoot–metatarsus, and metatarsus–hallux segments (Figure 2C). After marker placement, participants performed several familiarization trials to adapt to the laboratory environment and task requirements.

**Figure 2:**
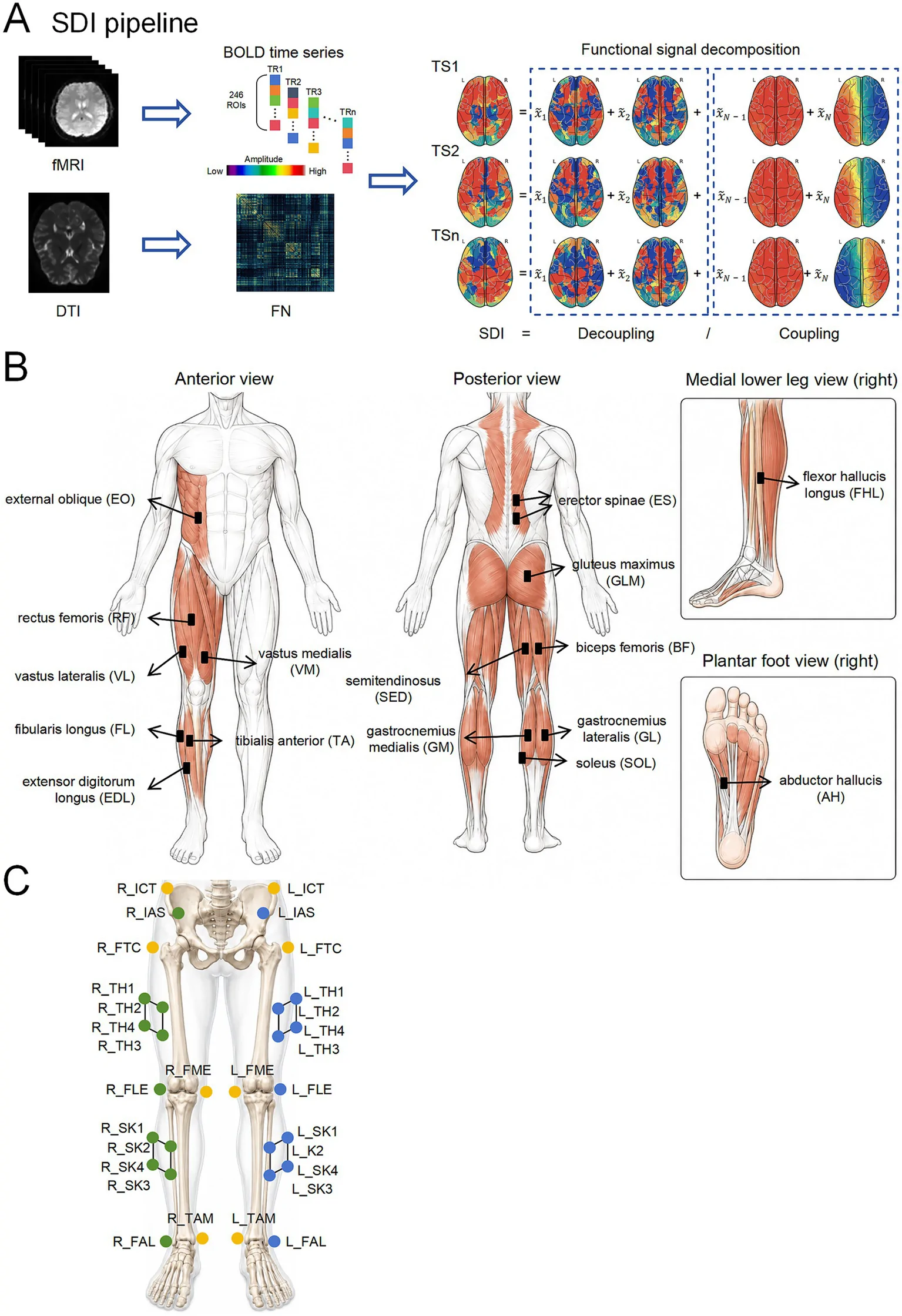
fMRI, DTI and surface EMG configuration. **(A)** Pipeline for structure–function decoupling analysis. Resting-state fMRI time series and DTI-derived structural matrices were constructed using the same brain atlas. Functional signals were projected onto structural connectome harmonics ordered from low to high graph frequency. For each participant, the cutoff was the first harmonic index at which cumulative graph-spectral energy reached at least 50% of total energy. Modes below and above this median-energy cutoff defined the structurally coupled and decoupled components, respectively, and SDI was calculated as the ratio of their regional signal norms across time. **(B)** Surface EMG sensor placement. Electrodes were placed on the muscle bellies of 16 muscles, including EO, ES, GLM, RF, VM, VL, SED, BF, TA, FL, EDL, GM, GL, SOL, FHL, and AH. The right lower limb was used for EMG recording. **(C)** Motion capture marker placement. Reflective markers were placed according to the CAST lower-limb model and the IOR multi-segment foot model. Markers were attached to anatomical landmarks including the anterior superior iliac spine, posterior superior iliac spine, medial and lateral femoral epicondyles, proximal fibular head, tibial tuberosity, medial and lateral malleoli, posterior calcaneus, proximal posterior calcaneus, first, second, and fifth metatarsal heads, base of the fifth metatarsal, proximal medial phalanx of the hallux, bases of the first and second metatarsals, sustentaculum tali, peroneal tubercle, and navicular tuberosity. In addition, thigh and shank cluster markers were placed at the mid-thigh and mid-shank segments bilaterally.

All biomechanical tests were performed barefoot. For the gait task, participants were instructed to walk naturally at a self-selected comfortable speed along a walkway containing embedded plantar pressure/force platforms (Figure 1C). They were asked to look forward, maintain a natural arm swing, and step on the effective measurement area of the platform during walking. Three walking trials were collected for each participant.

For the sit-to-stand (STS) task, participants performed repeated stand–sit–stand transitions while both feet remained on the plantar pressure/force platform (Figure 1D). This setup allowed continuous recording of center of pressure and ground reaction force signals throughout the postural transition. Each participant completed three STS trials.

The flow of the experimental visit is shown in Figure 1E. After MRI scanning, participants underwent electrode and marker placement, followed by pre-experiment training. After familiarization with the walking and stand/sit tasks, signal quality was checked, and formal synchronized data collection was then performed. Motion capture, plantar pressure, ground reaction force, and sEMG signals were recorded simultaneously during both gait and STS tasks. The timeline of the biomechanical testing session is shown in Figure 1F. After preparation, participants completed three gait trials and three STS trials, and the whole biomechanical testing session lasted approximately 30 min.

### MRI acquisition and preprocessing

MRI data were acquired before biomechanical testing (Figure 1A). The MRI protocol included resting-state fMRI and DTI. During rs-fMRI scanning, participants were instructed to remain awake, minimize head movement, and avoid systematic thinking.

MRI data were acquired from 29 participants using a 3-T Siemens Verio scanner. High-resolution T1-weighted structural images were acquired using a three-dimensional magnetization-prepared rapid gradient-echo (MPRAGE) sequence with the following parameters: repetition time (TR) = 2,000 ms, echo time (TE) = 2.57 ms, inversion time (TI) = 900 ms, flip angle = 9°, matrix = 256 × 256, reconstructed voxel size = 0.977 × 0.977 × 1.0 mm^3^, and GRAPPA acceleration factor = 2. Resting-state functional MRI (rs-fMRI) data were acquired using a two-dimensional echo-planar imaging (EPI) sequence with the following parameters: TR = 2,250 ms, TE = 30 ms, flip angle = 90°, matrix = 72 × 72, in-plane resolution = 3.47 × 3.47 mm^2^, 33 slices, slice thickness = 3.5 mm, and slice spacing (center-to-center) = 4.2 mm. A total of 200 functional volumes were acquired, corresponding to a nominal acquisition duration of 450 s. Diffusion-weighted imaging (DWI) data were acquired using an EPI sequence with the following parameters: TR = 10,500 ms, TE = 95 ms, flip angle = 90°, matrix = 128 × 128, in-plane resolution = 1.95 × 1.95 mm^2^, slice thickness = 2.0 mm, slice spacing (center-to-center) = 2.6 mm, and GRAPPA acceleration factor = 2. Thirty distinct diffusion-encoding directions at b = 1,000 s/mm^2^ were acquired with three repetitions, yielding 90 diffusion-weighted volumes. An additional 12 non-diffusion-weighted (b = 0 s/mm^2^) volumes were acquired, resulting in a total of 102 volumes and a nominal acquisition duration of 1,071 s.

Resting-state fMRI data were preprocessed with fMRIPrep and normalized to MNI152NLin2009cAsym space at 2-mm isotropic resolution. The Brainnetome Atlas was resampled to each participant’s preprocessed BOLD image using nearest-neighbor interpolation, and regional time series were extracted from 246 ROIs. Nuisance regression included a 24-parameter motion model, white matter and cerebrospinal fluid signals, motion outlier regressors, an intercept, and a linear trend. The residuals were band-pass filtered at 0.01–0.08 Hz and z-standardized. Framewise displacement (FD) was derived from fMRIPrep confounds, and volumes with FD > 0.5 mm were censored before functional connectivity and structure–function decoupling index estimation. All participants retained at least 80 time points after censoring. Mean FD was compared between groups and included in MRI sensitivity analyses where specified.

Diffusion MRI data were preprocessed with QSIPrep for eddy-current-induced distortion and head-motion correction, b-vector rotation, brain-mask generation, and transformation to ACPC-aligned diffusion space. Whole-brain probabilistic tractography was performed in MRtrix3. The Brainnetome Atlas was transformed to each participant’s diffusion space, and fiber orientation distributions were estimated using constrained spherical deconvolution. Approximately 2 million streamlines were reconstructed using iFOD2 with dynamic seeding. Structural connectivity matrices were generated with tck2connectome; edge weights were defined as streamline counts between ROI pairs, and matrices were symmetrized with diagonal elements set to zero.

### FN-SDI calculation and network-level analysis

For structure–function decoupling, resting-state BOLD time series were extracted for each ROI (Sun et al., 2024; Zhang et al., 2024). The fMRI–DTI framework followed graph signal processing principles (Figure 2A). The fiber-number (FN) structural connectivity matrix was denoted as *W*. The normalized graph Laplacian *L* was computed as:

$$L=I-D^{-1/2}WD^{-1/2}$$ (1)

where I is the identity matrix and D is the degree matrix of W. Eigendecomposition of L yielded the structural graph harmonic basis U, with harmonics ordered by increasing Laplacian eigenvalue (graph frequency). The regional BOLD signal matrix X was projected onto this basis using the graph Fourier transform, $\hat{X}=U^{T}X$ . For each participant, the energy of harmonic k was calculated as the mean squared graph-Fourier coefficient across time, $E_{k}\;=\;\;\frac{1}{T}\sum_{t=1}^{T}{\hat{X}}_{k}{\left(t\right)}^{2}$ . The cutoff C was defined as the smallest index for which cumulative energy reached at least half of the total spectral energy: $C=\;\;min\left\{\mathrm{c:}\sum_{\mathrm{k=1}}^{c}E_{k}\;\geq \;\;0\mathrm{.5}\;\sum_{\mathrm{k=1}}^{N}E_{k}\right\}$ . Harmonics 1 through C were assigned to the low-frequency, structurally coupled component, and harmonics C + 1 through N were assigned to the high-frequency, structurally decoupled component. This participant-specific median-energy split follows the equal-energy principle of the original SDI framework. It provides a data-adaptive partition when no canonical graph-frequency boundary exists, avoids an arbitrary split by the number of harmonics when spectral energy is concentrated at lower graph frequencies, and prevents either side of the SDI ratio from being favored solely by a gross imbalance in retained signal energy. The 50% criterion is therefore an operational spectral definition rather than a sharp biological boundary (Preti and Van De Ville, 2019).

For each ROI, the FN-based structure–function decoupling index (FN-SDI) was calculated as:

$$FN-SDI_{i}=\frac{{\left\|X_{high,i}\right\|}_{2}}{{\left\|X_{low,i}\right\|}_{2}}$$ (2)

where i denotes the ROI, X_high is the decoupled component, and X_low is the coupled component. Higher FN-SDI indicates stronger decoupling from structural connectivity. Log₂-transformed FN-SDI values were used for group-level visualization.

ROI-wise FN-SDI values were summarized into ten networks: cortical ROIs mapped to Yeo 7-network parcellation (visual, somatomotor, dorsal attention, ventral attention, limbic, frontoparietal, default mode) and subcortical ROIs grouped anatomically into amygdala/hippocampus, basal ganglia/striatum, and thalamus. Network-level FN-SDI was calculated as the mean of constituent ROIs. Between-group differences were assessed using Welch’s t-test with Benjamini–Hochberg FDR correction.

At the regional level, age-group differences in log_2_-SDI were tested separately for all 246 Brainnetome atlas ROIs using two-sided Welch’s t-tests. The resulting 246 raw *p* values were treated as a single testing family and adjusted using the Benjamini-Hochberg false-discovery-rate procedure. A regional age-group difference was considered significant at q_FDR < 0.05.

### Surface EMG preprocessing

Raw sEMG signals were visually inspected to remove trials with prominent artifacts. For muscle synergy extraction, the signals were band-pass filtered (20–450 Hz, 4th-order zero-phase Butterworth), full-wave rectified, and low-pass filtered at 4–10 Hz to obtain the linear envelope (De Luca et al., 2010). For intermuscular coherence (IMC) analysis, a completely separate preprocessing stream was utilized to preserve oscillatory features: the raw signals were only high-pass filtered at 20 Hz to remove baseline drift and demeaned. These unrectified signals were directly utilized for subsequent frequency-domain coupling estimations. For gait, gait events were identified from the synchronized biomechanical recordings and used to segment each gait cycle into seven predefined phases. For IMC analysis, phase-specific segments of the unrectified EMG signals were retained at the native sampling rate (2,000 Hz) and analyzed in the original time domain before gait-cycle time normalization. Time normalization to 0–100% of the gait cycle was applied only for temporal representation and analyses requiring a normalized cycle and was not applied before coherence estimation. For sit-to-stand (STS) transitions, EMG signals were segmented into four task-specific phases based on force plate data, motion capture, and temporal events: (1) flexion momentum, (2) momentum transfer, (3) extension, and (4) stabilization (Clark et al., 2010). For muscle synergy analysis, EMG envelopes were normalized to each participant’s peak amplitude across valid trials. This amplitude normalization was not applied to the unrectified EMG signals used for IMC estimation.

### Hybrid muscle-kinematic synergy extraction

To capture the integrated coordination of muscles and joints, we performed enhanced hybrid synergy extraction using non-negative matrix factorization (NMF) (Scano et al., 2022). Time-aligned EMG envelope data (*E*) and joint kinematics (*D*) were first scaled to eliminate unit differences using the Frobenius norm:

$$E^{\prime }=E\frac{target}{{\left\|E\right\|}_{F}}\;,\;\;D^{\prime }=D\frac{target}{{\left\|D\right\|}_{F}}$$ (3)

where $target={\left\|E\right\|}_{F}+{\left\|D\right\|}_{F},$ and ${\left\|\cdot \right\|}_{F}$ is the Frobenius norm. A weighted concatenation was applied to form the hybrid data matrix:

$$M=\left[\omega_{\mathrm{EMG}}E^{\prime };\;\omega_{\mathrm{DOF}}D^{\prime }\right]$$ (4)

where default weights $\omega_{\mathrm{EMG}}=\omega_{\mathrm{DOF}}=1.$ NMF was then applied to decompose *M* as (Olikkal et al., 2022):

$$M\approx W\;H+E_{r}$$ (5)

Here, *W* represents the spatial composition of the hybrid modules (muscle + joint contributions), *H* represents temporal activation coefficients, and $E_{r}$ is the residual matrix. The number of synergies was determined using variance accounted for (VAF ≥ 95%):

$$VAF=1-\frac{{\left\|M-WH\right\|}_{F}^{2}}{{\left\|M\right\|}_{F}^{2}}$$ (6)

Muscle synergy similarity was calculated to evaluate the extent to which an individual’s spatial muscle coordination pattern deviated from a healthy young baseline. To rigorously prevent the artificial inflation of similarity scores within the young group, a leave-one-out cross-validation (LOOCV) approach was utilized. Specifically, for each young participant, the reference template was generated by averaging the spatial synergy matrices (W) of the remaining young participants. For older adults, the reference template was derived from the average of all young participants. The similarity between an individual’s spatial synergy matrix and the corresponding reference template was exclusively quantified using Pearson’s correlation coefficient.

### Intermuscular coherence analysis

Intermuscular coherence (IMC) was used to quantify frequency-domain coupling between pairs of muscles. For gait, coherence was estimated from the unrectified EMG signals at the native sampling rate of 2,000 Hz before gait-cycle time normalization. Gait events identified from the synchronized biomechanical recordings were used to segment each gait cycle into seven predefined phases. For each participant and each phase, the corresponding unrectified EMG segments from all valid gait cycles across the three trials were concatenated in chronological order for each muscle, thereby forming a phase-specific time series for subsequent coherence estimation. For STS, the unrectified EMG signals were similarly segmented according to the four predefined task phases, and the corresponding segments from all valid trials were concatenated within each phase. Thus, time normalization was not applied to the EMG signals used for coherence estimation.

Magnitude-squared coherence between two EMG signals, x and y , was calculated as:

$$C_{xy}\left(f\right)=\frac{{\left|P_{xy}\left(f\right)\right|}^{2}}{P_{xx}\left(f\right)P_{yy}\left(f\right)}$$ (7)

where $P_{xy}\left(f\right)$ denotes the cross-spectral density between signals x and y , $P_{xx}\left(f\right)$ and $P_{yy}\left(f\right)$ denote their respective autospectral densities. Spectral densities were estimated using Welch’s method with Hamming windows and 50% overlap. For each participant and movement phase, the window length was set to the nearest power of two corresponding to approximately one-eighth of the total length of the concatenated phase-specific signal, with a minimum window length of 128 samples (64 ms at the 2,000-Hz sampling rate). This procedure allowed the spectral estimation parameters to accommodate differences in the amount of available phase-specific data across participants and movement phases.

Coherence was calculated for all pairwise combinations of the 16 recorded muscles. For each muscle pair, coherence values were averaged within the 15–35 Hz beta band, which is commonly interpreted as reflecting shared neural input to the involved muscles (Conway et al., 1995), to obtain beta-band IMC. Phase-specific 16 × 16 symmetric coherence matrices were then constructed for each participant during gait and STS. Mean IMC was calculated as the average beta-band coherence across all unique muscle pairs within each task or phase, as appropriate. In addition, graph-theoretical network efficiency was calculated from the corresponding muscle coherence networks to characterize the efficiency of information integration across the neuromuscular system. Phase-specific between-group differences in IMC connectivity were subsequently assessed using network-based statistics (NBS), as described below.

### COP and GRF processing

COP trajectories were extracted from plantar pressure/force platform data (9260AA6, Kistler, Switzerland). For gait, COP-related indicators included anteroposterior COP displacement (COP-AP), mediolateral COP displacement (COP-ML), and total COP path length (COP-TL). Vertical ground reaction force curves were normalized to body weight and gait cycle duration. The first GRF peak (GRF-P1) and second GRF peak (GRF-P2) were extracted to characterize loading response and push-off capacity, respectively.

For STS, COP indicators included anterior COP displacement (COP-A), posterior COP displacement (COP-P), and COP-TL. GRF and temporal parameters included maximum GRF (GRF-Max), movement time (MT), and time to seat-off (T-SO). These variables were used to evaluate weight transfer, dynamic postural stability, lower-limb force generation, and postural transition efficiency.

### Multimodal association analysis

Regional SDI values were defined according to the Brainnetome 246-region atlas. Candidate ROIs for the SDI–muscle synergy and SDI–IMC association analyses were specified in two steps. First, we retained the nine ROIs showing significant young-versus-older differences in SDI after Benjamini–Hochberg false-discovery-rate (FDR) correction across all 246 ROIs (qFDR < 0.05; ROI168, ROI174, ROI176, ROI180, ROI188, ROI195, ROI219, ROI220, and ROI226). Second, the bilateral paracentral-lobule lower-limb area 4 regions (ROI067 and ROI068) were included *a priori* on the basis of their anatomical relevance to lower-limb motor control. ROI selection was performed independently of muscle synergy similarity, IMC values, and the corresponding cross-modal association statistics.

Associations of regional SDI with muscle synergy similarity and mean IMC were subsequently examined separately for gait and STS. To distinguish cross-modal associations from differences attributable primarily to the separation between the young and older groups, multiple linear regression models were fitted with regional SDI as the dependent variable, the corresponding neuromuscular metric as the predictor, and age-group membership (young vs. older) as a covariate. Continuous predictor and outcome variables were standardized before model fitting, and standardized regression coefficients (β) with 95% confidence intervals (CIs) were reported. For each association family, Benjamini–Hochberg FDR correction was applied across the 11 candidate ROIs separately for gait and STS. Because the candidate ROIs were partly identified from age-group differences in the same cohort, these cross-modal analyses were considered exploratory despite multiplicity correction. For associations showing nominal significance in the group-adjusted models, group-stratified analyses were additionally performed in young and older adults separately and visualized using scatter plots with group-specific linear regression lines. Slopes, *p* values, and coefficients of determination (r^2^) were reported for these descriptive analyses. These stratified analyses were exploratory and were used to characterize within-group association patterns rather than to establish statistically significant differences in slopes between age groups.

### Statistical analysis

Continuous variables were assessed for normality. Normally distributed data are presented as mean ± standard deviation (SD), whereas non-normally distributed variables are presented as median and interquartile range, as appropriate. Between-group comparisons were performed using independent-samples t-tests or Mann–Whitney U tests according to data distribution, and categorical variables were compared using chi-square or Fisher’s exact tests.

ROI-level age-group differences in SDI were assessed using two-sided Welch’s t-tests across all 246 Brainnetome regions, with the complete set of 246 *p* values corrected using the Benjamini–Hochberg FDR procedure. Network-level SDI comparisons were similarly corrected across the ten predefined networks. For IMC connectivity matrices, between-group differences were assessed using network-based statistics (NBS; 5,000 permutations; component-forming threshold, uncorrected *p* < 0.05; FWE-corrected *p* < 0.05). NBS analyses were conducted separately for each movement phase; therefore, the phase-specific NBS findings were considered exploratory with respect to comparisons across phases. Group differences in muscle synergy similarity, mean IMC, IMC network efficiency, and predefined COP/GRF outcomes were assessed using independent-samples t-tests or Mann–Whitney U tests, as appropriate. Cross-modal associations between regional SDI and muscle synergy similarity or mean IMC were evaluated using multiple linear regression adjusted for age-group membership. Continuous predictors and outcomes were standardized before analysis, and standardized regression coefficients (β) with 95% CIs were reported. Benjamini–Hochberg FDR correction was applied across the 11 candidate ROIs separately for each task and neuromuscular metric. Associations with uncorrected *p* < 0.05 but qFDR ≥ 0.05 were described as nominally significant and interpreted as exploratory. Group-stratified simple linear regression analyses were performed for selected nominal associations to visualize within-group patterns in young and older adults separately. A difference in statistical significance between the two groups was not interpreted as evidence of a between-group difference in slopes. All tests were two-sided, and *p* < 0.05 or qFDR < 0.05, as applicable, was considered statistically significant.

## Results

### Participant characteristics

A total of 36 participants were assessed for eligibility, and 29 participants were included in the final analysis, including 13 young adults and 16 older adults. Seven participants were excluded because they did not meet the inclusion criteria, declined to participate, or had other reasons for exclusion. All enrolled participants completed gait analysis, plantar pressure/ground reaction force recording, sEMG acquisition, resting-state fMRI, and DTI scanning. The participant flow diagram is shown in Figure 3.

**Figure 3:**
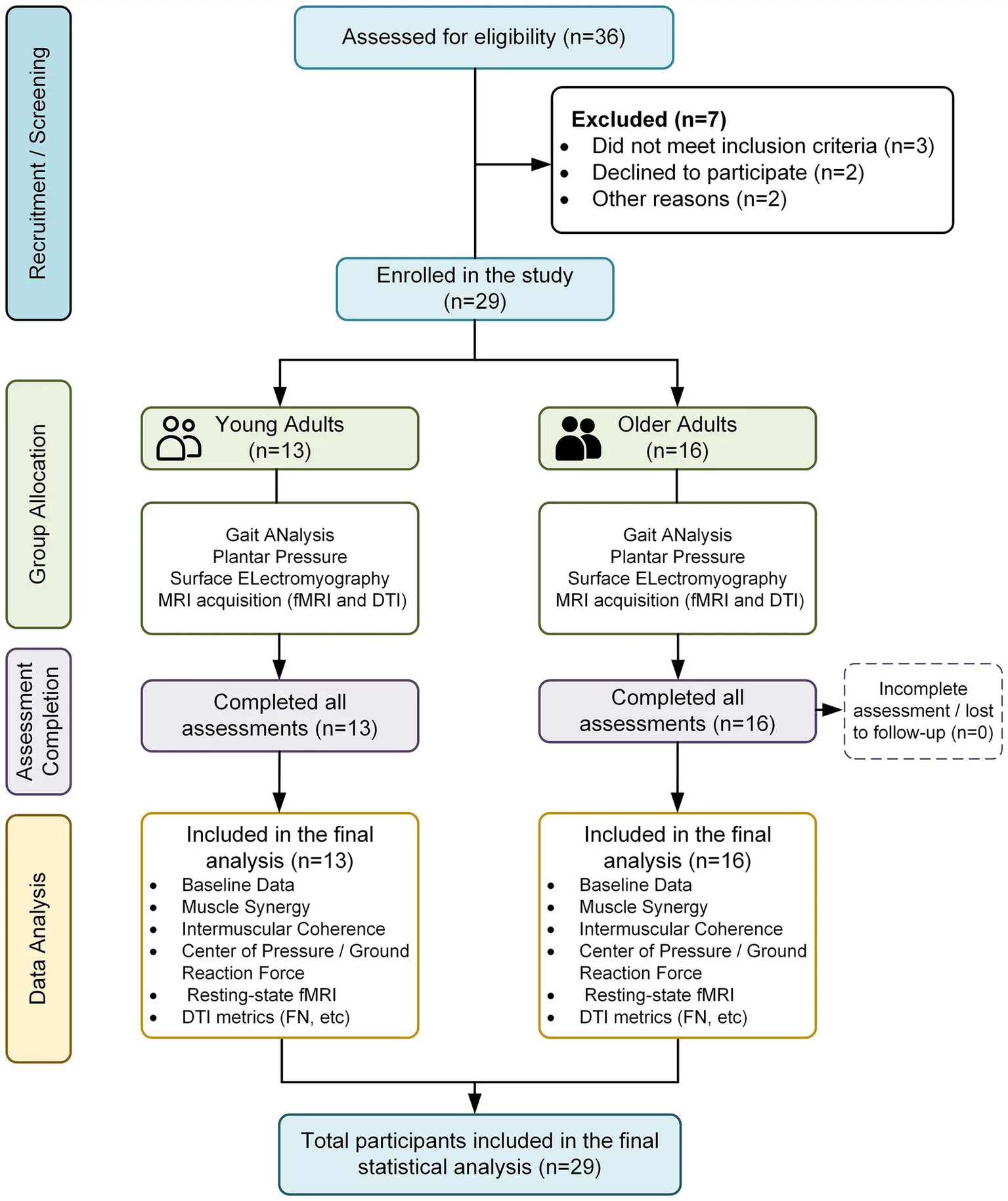
Participant flow diagram of the clinical study.

The young group included 7 males and 6 females, and the older group included 8 males and 8 females. Height, weight, and BMI did not differ significantly between groups, indicating that the two groups were broadly comparable in basic anthropometric characteristics except for age (Table 1).

**Table 1:** Participant demographics.

Characteristics	Young group	Older group	*p* value
Sex (male/female)	7/6	8/8	NA
Age (years)	24.08 ± 2.5	71.88 ± 3.67	< 0.0001
Height (cm)	169.7 ± 7.66	164.6 ± 6.96	0.0732
Weight (kg)	65.31 ± 23.90	63.22 ± 8.14	0.7454
BMI (kg/m^2^)	22.42 ± 6.85	23.52 ± 2.51	0.5571
Gait speed (m/s)	1.038 ± 0.08	1.047 ± 0.14	0.8440

*p* > 0.05 indicates no significant between-group difference. Data are presented as mean ± SD.

### Age-related differences in brain structure–function decoupling

Brain structure–function decoupling was quantified using the log2-transformed structure–function decoupling index (log2-SDI), derived from resting-state fMRI signals and DTI-based structural connectivity. Cortical and subcortical surface maps revealed distinct spatial distributions of mean log2-SDI in young and older adults (Figure 4A). At the global level, older adults showed a lower whole-brain mean log2-SDI than young adults (Figure 4B; *p* = 0.031, Cohen’s d = −0.82), indicating a modest reduction in functional decoupling from the underlying structural connectome with aging.

**Figure 4:**
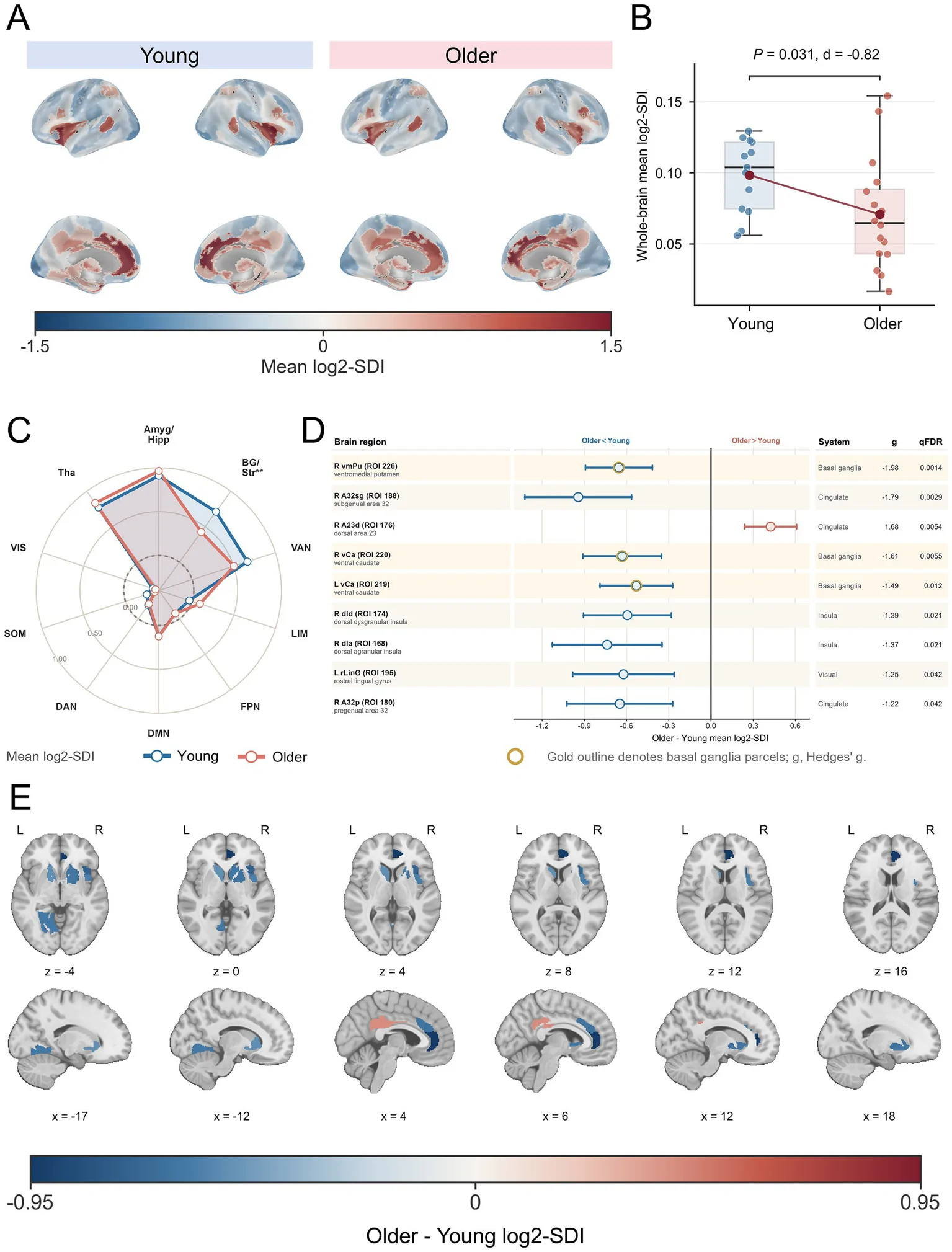
Age-related differences in brain structure–function decoupling. **(A)** Cortical and subcortical surface maps showing mean log2-transformed structure–function decoupling index (log2-SDI) in young and older adults. **(B)** Whole-brain mean log2-SDI comparison between groups. **(C)** Network-level comparison of mean log2-SDI across ten large-scale networks. **(D)** Forest plot showing the nine brain regions that survived Benjamini-Hochberg false-discovery-rate correction across all 246 Brainnetome ROIs (q_FDR < 0.05). The plotted points represent the mean between-group difference in log2-SDI calculated as older minus young, and the horizontal lines indicate the corresponding 95% confidence intervals (CIs). Negative values therefore indicate lower SDI in older adults, whereas positive values indicate higher SDI in older adults. The corresponding standardized effect size, Hedges’ g, is reported separately in the right-hand column. Blue markers indicate lower SDI in older adults, red markers indicate higher SDI in older adults, and gold outlines denote basal ganglia parcels. **(E)** Axial and sagittal maps showing the ROIs that survived the same whole-brain FDR criterion; blue indicates lower SDI and red indicates higher SDI in older adults.

To determine whether this global alteration was driven by specific functional systems, mean log2-SDI was further compared across ten large-scale brain networks. Among these networks, only the basal ganglia/striatal network showed a significant group difference (Figure 4C), suggesting that age-related SDI alterations were not uniformly distributed across the brain but were preferentially localized to subcortical motor-control systems.

ROI-wise analysis identified nine regions with significant between-group differences in log2-SDI after Benjamini-Hochberg FDR correction across all 246 Brainnetome ROIs (q_FDR < 0.05; Figure 4D). Most of these regions showed lower SDI in older adults, including the right ventromedial putamen, bilateral ventral caudate nuclei, right dorsal dysgranular insula, right dorsal agranular insula, right subgenual anterior cingulate cortex, right pregenual anterior cingulate cortex, and left rostral lingual gyrus. In contrast, the right dorsal area 23 region showed higher SDI in older adults. The forest plot further demonstrated that the most robust age-related SDI differences were concentrated in basal ganglia, cingulate, and insular regions.

Axial and sagittal maps of older-minus-young log2-SDI differences confirmed the spatial distribution of these regional alterations (Figure 4E). Blue regions indicate lower SDI in older adults, whereas red regions indicate higher SDI in older adults. Overall, these findings suggest that healthy aging is associated with regionally specific changes in brain structure–function decoupling, mainly involving networks related to motor automaticity, cognitive–motor regulation, and sensory/interoceptive integration, rather than isolated alterations in the primary motor cortex.

### Sensitivity analysis of continuous age effects on MRI SDI

Age was nearly collinear with age-group membership (point-biserial r = 0.992), with a 37-year gap between the oldest young participant and the youngest older participant. In pooled analyses, age was negatively associated with whole-brain mean log2-SDI (Pearson r = −0.372, *p* = 0.047) and basal ganglia/striatum network log2-SDI (Pearson r = −0.653, *p* < 0.001). However, after separating the between-group contrast from within-group age variation, the within-group-centered age term was not associated with whole-brain SDI (standardized β = 0.106, 95% CI -0.437 to 0.649, *p* = 0.702, FDR q = 0.702) or basal ganglia/striatum SDI (standardized β = −0.233, 95% CI -0.650 to 0.185, *p* = 0.275, FDR q = 0.549). The same conclusion was obtained after adjustment for sex, mean framewise displacement, and in the motion-QC subset. Thus, the pooled associations primarily reflected differences between the two age groups rather than a demonstrable continuous age effect within the sampled age ranges.

### Age-related differences in muscle synergy organization

The number of extracted muscle synergy modules differed by task. During gait, young and older adults showed comparable numbers of muscle synergy modules, suggesting that the basic dimensionality of gait-related modular control was largely preserved with aging. In contrast, during the STS task, older adults exhibited a significantly greater number of extracted muscle synergy modules than young adults (*p* = 0.0164; Figure 5A). This indicates that aging may increase the dimensionality or fragmentation of neuromuscular control during non-cyclic postural transitions.

**Figure 5:**
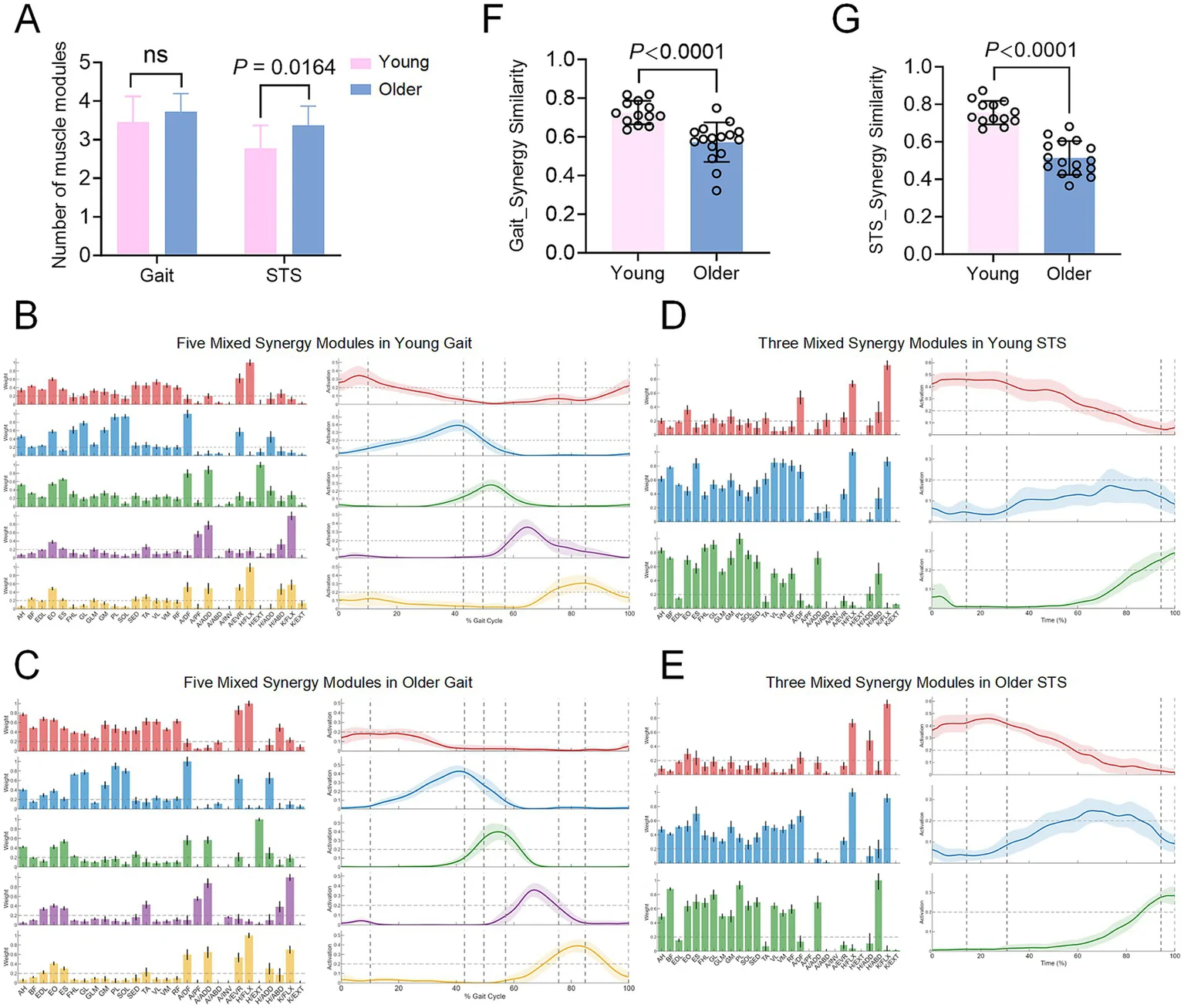
Muscle synergy similarity during gait and sit-to-stand tasks. **(A)** Comparison of the number of extracted muscle synergy modules between young and older adults during gait and sit-to-stand (STS) tasks. **(B,C)** Muscle-joint hybrid synergy patterns during gait in young and older adults, respectively. Each row represents one synergy module. Bar plots show the spatial weights of muscles and joint degrees of freedom, and the corresponding curves show the temporal activation profiles across the normalized gait cycle. Five modules were extracted in both groups during gait. **(D,E)** Muscle-joint hybrid synergy patterns during the STS task in young and older adults, respectively. Three modules were extracted in both groups. Bar plots represent the spatial weights of muscles and joint degrees of freedom, and the corresponding curves represent the temporal activation profiles across the normalized STS cycle. **(F,G)** Comparison of muscle synergy similarity between young and older adults during gait **(F)** and STS **(G)**. Individual data points are overlaid on the group summaries. Data are presented as mean±SD. ns, not significant; STS, sit-to-stand.

Hybrid muscle–joint synergy analysis further showed that both groups exhibited five modules during gait and three modules during STS (Figures 5B–E). During gait, young adults displayed phase-specific neuromechanical modules corresponding approximately to early stance, mid-stance, late stance, pre-swing/early swing, and late swing. Older adults retained a five-module structure but showed more distributed muscle and joint weighting patterns, suggesting broader muscle recruitment and compensatory involvement of ankle-foot and hip-trunk components. During STS, both groups showed three hybrid modules corresponding to early postural preparation, mid-phase weight transfer, and late-phase standing stabilization. Older adults showed broader contributions from trunk, quadriceps, hamstrings, calf, and intrinsic foot muscles, suggesting a more distributed and compensatory neuromechanical strategy during postural transition.

Muscle synergy similarity was significantly lower in older adults than in young adults during both gait and STS (both *p* < 0.0001; Figures 5F,G). These findings indicate greater deviation from the young-reference neuromuscular coordination pattern in older adults across both locomotor and postural-transition tasks.

### Associations between muscle synergy similarity and SDI

We next examined associations between muscle synergy similarity and SDI across the 11 candidate ROIs using multiple linear regression adjusted for age-group membership (Figure 6). During gait, none of the regional associations reached nominal significance after adjustment for age group (Figure 6A).

**Figure 6:**
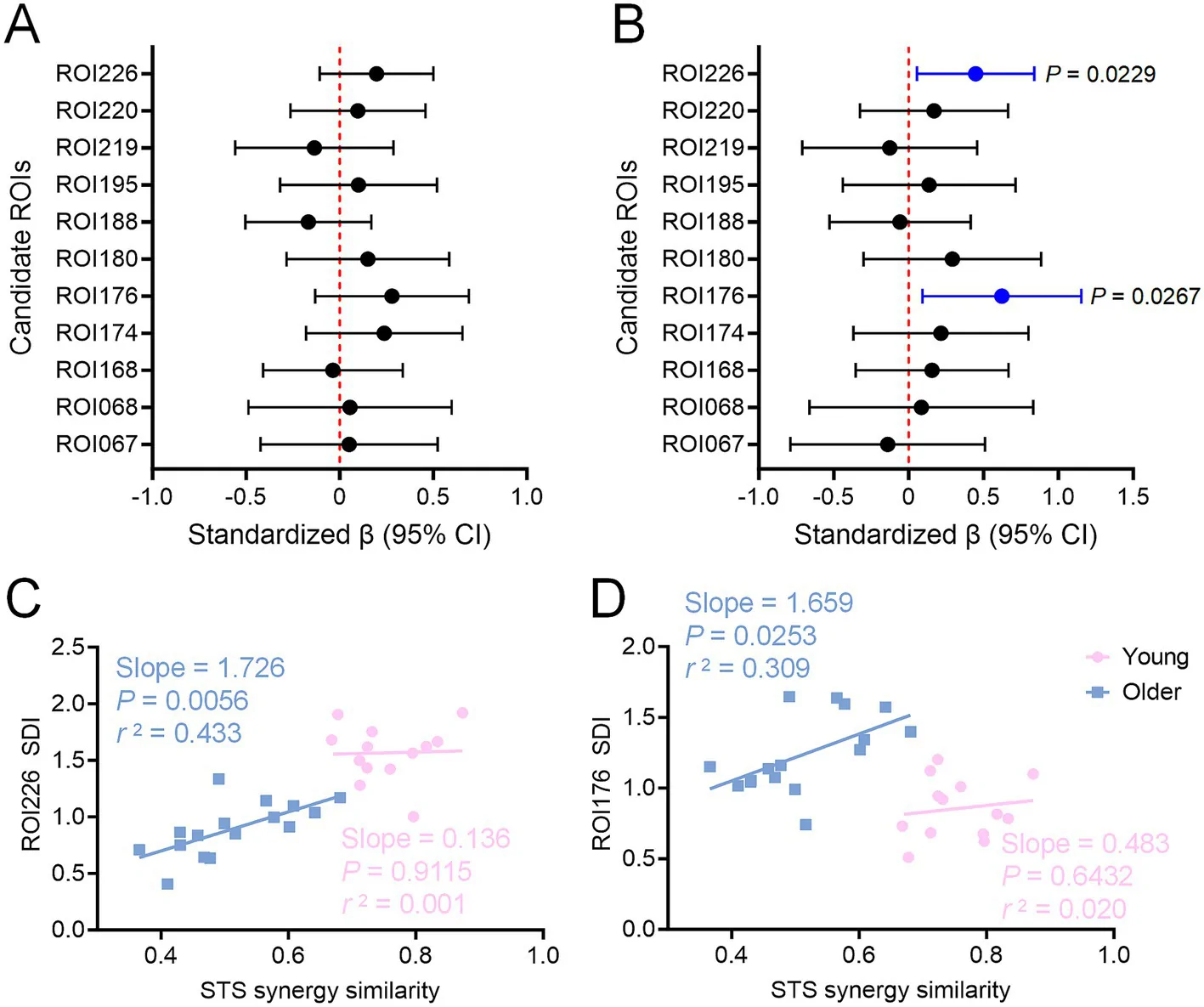
Associations between muscle synergy similarity and regional structure–function decoupling index (SDI) during gait and sit-to-stand tasks. **(A,B)** Forest plots showing associations between muscle synergy similarity and SDI across the 11 candidate regions of interest (ROIs) during gait **(A)** and sit-to-stand (STS) **(B)**. Multiple linear regression models were adjusted for age-group membership (young vs. older). Points represent standardized regression coefficients (β), and horizontal error bars indicate 95% confidence intervals (CIs); the red dashed line denotes β = 0. **(C,D)** Group-stratified scatter plots illustrating the associations of STS muscle synergy similarity with SDI in ROI226 **(C)** and ROI176 **(D)**. Individual participants are shown separately for young and older adults, with group-specific linear regression lines. Slope, *p* value, and r^2^ are reported for each group. These stratified analyses were used to visualize within-group association patterns and were considered exploratory. ROI226, right ventromedial putamen; ROI176, right dorsal area 23.

During STS, muscle synergy similarity showed nominal positive associations with SDI in the right ventromedial putamen (ROI226; standardized β = 0.448, 95% CI 0.056 to 0.84, *p* = 0.0229) and right dorsal area 23 (ROI176; β = 0.623, 95% CI 0.093 to 1.153, *p* = 0.0267) (Figure 6B). Neither association survived Benjamini–Hochberg FDR correction across the 11 candidate ROIs. These associations were therefore interpreted as nominal and exploratory rather than confirmatory.

Group-stratified analyses were subsequently used to visualize the within-group patterns underlying these two STS associations (Figures 6C,D). For ROI226, STS synergy similarity was positively associated with SDI in older adults, whereas no corresponding association was observed in young adults. For ROI176, a positive association was also observed in older adults, but not in young adults. Because these stratified analyses were exploratory and the sample sizes within each group were small, the apparent differences in within-group significance were not interpreted as evidence of statistically different slopes between age groups.

### Age-related differences in IMC during gait and STS

During gait, phase-specific IMC analysis revealed distinct between-group differences across the seven gait phases (Figure 7A). The most prominent difference was observed in Phase 3, in which a distributed network of muscle-muscle connections survived NBS correction. Because the matrices represent older-minus-young differences, the predominantly negative values within the significant component indicate lower IMC in older adults during this phase.

**Figure 7:**
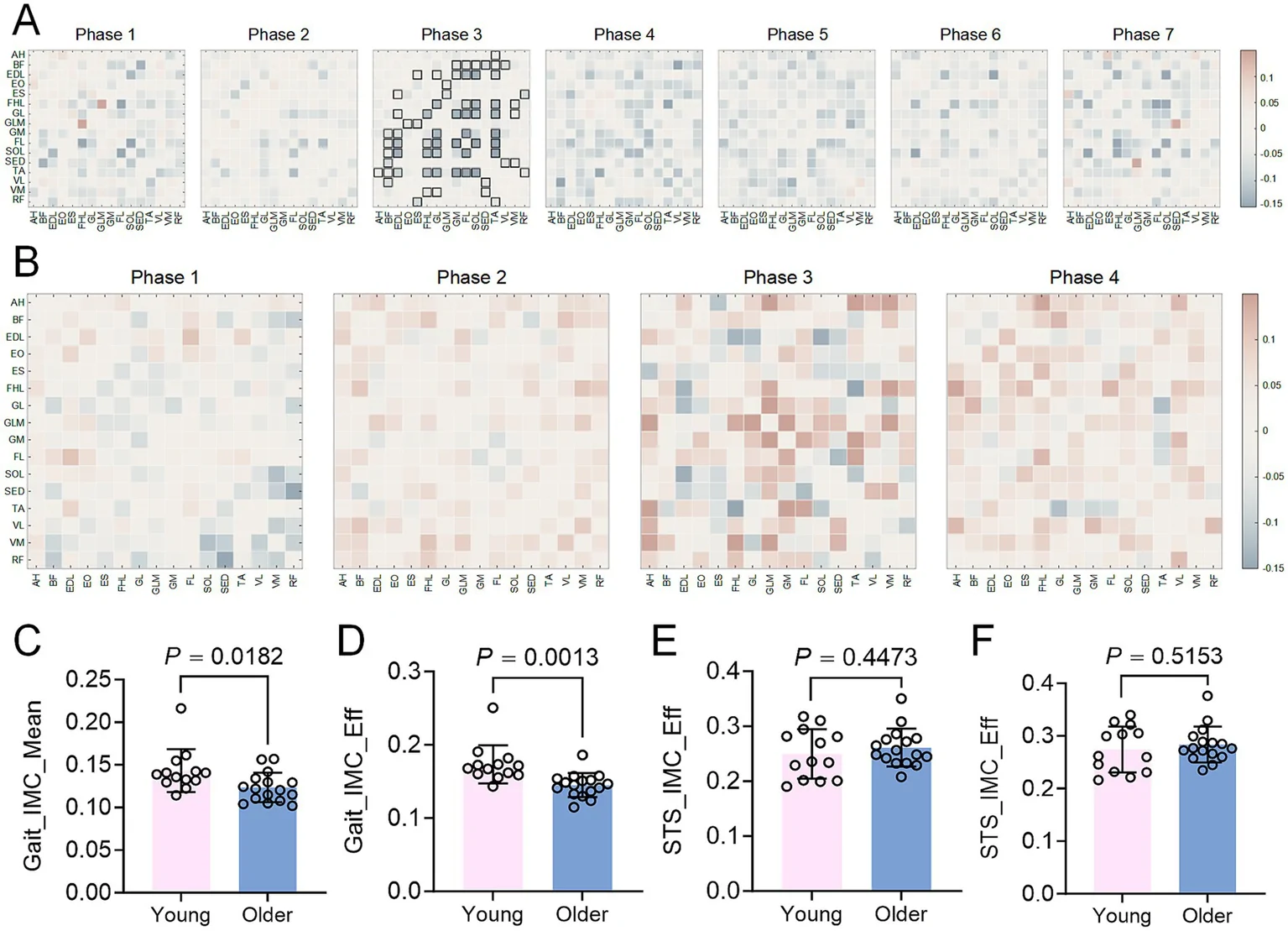
Age-related differences in intermuscular coherence during gait and sit-to-stand tasks. **(A)** Matrix plots showing between-group differences in IMC during gait across seven gait phases. Each matrix represents one phase of the normalized gait cycle, and each cell represents the coherence difference between one pair of muscles. The color scale indicates the difference between older adults and young adults, with red indicating higher IMC in older adults and blue indicating lower IMC in older adults. Black boxes indicate connections that survived NBS correction. **(B)** Matrix plots showing between-group differences in IMC during the sit-to-stand (STS) task across four task phases. **(C,D)** Comparisons of global IMC metrics between young and older adults during gait, including mean IMC **(C)** and IMC network efficiency **(D)**. **(E,F)** Comparisons of mean IMC **(E)** and IMC network efficiency **(F)** between young and older adults during STS. Individual data points are overlaid on the group summaries. Data are presented as mean±SD. IMC, intermuscular coherence; NBS, network-based statistics; STS, sit-to-stand. (Equations 1–7).

Consistent with the phase-specific findings, older adults showed significantly lower mean IMC (Figure 7C) and lower IMC network efficiency (Figure 7D) during gait than young adults. These results indicate reduced overall intermuscular coupling and network-level integration during walking in the older group.

During STS, between-group IMC differences were more spatially heterogeneous, and no NBS-significant muscle–muscle component was identified across the four task phases (Figure 7B). Neither mean IMC (Figure 7E) nor IMC network efficiency (Figure 7F) differed significantly between young and older adults. Together, these findings indicate a task-dependent pattern of age-group differences in intermuscular coherence, with more evident reductions during gait than during STS.

### Associations between IMC and SDI

We further examined associations between mean IMC and SDI across the same 11 candidate ROIs using multiple linear regression adjusted for age-group membership (Figure 8). During gait, none of the IMC-SDI associations reached nominal significance (Figure 8A).

**Figure 8:**
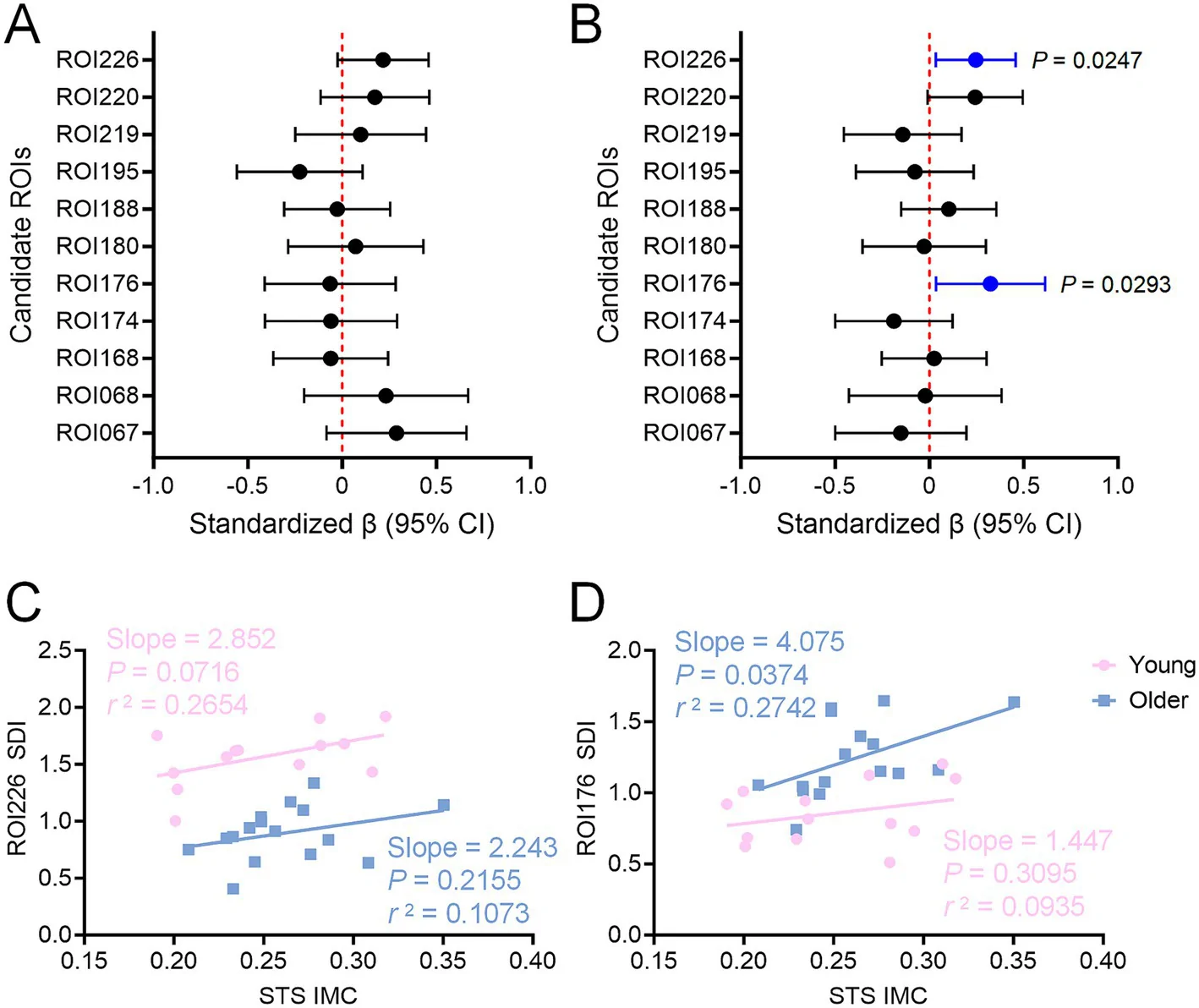
Associations between intermuscular coherence and regional structure–function decoupling index (SDI) during gait and sit-to-stand tasks. **(A,B)** Forest plots showing associations between mean intermuscular coherence (IMC) and SDI across the 11 candidate regions of interest (ROIs) during gait **(A)** and sit-to-stand (STS) **(B)**. Multiple linear regression models were adjusted for age-group membership (young vs. older). Points indicate standardized regression coefficients (β), and horizontal error bars represent 95% confidence intervals (CIs); the red dashed line denotes β = 0. **(C,D)** Group-stratified scatter plots illustrating the associations between STS IMC and SDI in ROI226 **(C)** and ROI176 **(D)**. Individual participants are shown separately for young and older adults, with group-specific linear regression lines. Slope, *p* value, and r^2^ are reported for each group. These stratified analyses were performed to visualize within-group association patterns and were considered exploratory. ROI226, right ventromedial putamen; ROI176, right dorsal area 23.

During STS, nominal positive associations were observed for the right ventromedial putamen (ROI226; standardized β = 0.246, 95% CI 0.034 to 0.457, *p* = 0.0247) and right dorsal area 23 (ROI176; standardized β = 0.324, 95% CI 0.035 to 0.614, *p* = 0.0293) (Figure 8B). However, neither association survived Benjamini–Hochberg FDR correction across the 11 candidate ROIs. Accordingly, these findings were considered nominal and exploratory.

Group-stratified analyses showed different within-group patterns for these two regions (Figures 8C,D). For ROI226, the association between STS IMC and SDI did not reach significance in either young adults or older adults. For ROI176, a positive within-group association was observed in older adults, whereas the association was not significant in young adults. These stratified results were considered descriptive and exploratory; differences in within-group statistical significance were not interpreted as evidence of different slopes between age groups.

### COP and GRF parameters during gait and STS

COP and GRF parameters are summarized in Figure 9 and Table 2. During gait, older adults showed significantly greater COP-AP, COP-ML, and COP-TL than young adults, indicating increased COP excursion and less efficient pressure-center control. In contrast, GRF-P1 and GRF-P2 were significantly lower in older adults, suggesting reduced loading response and push-off force generation during walking.

**Figure 9:**
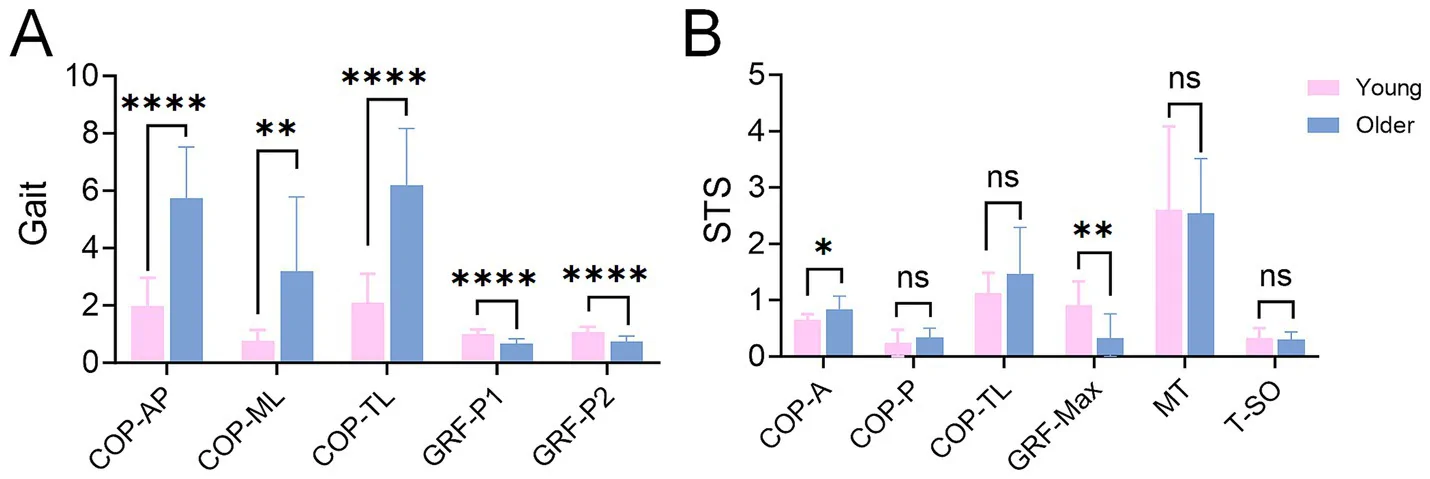
Center of pressure and ground reaction force parameters during gait and sit-to-stand tasks. **(A)** Comparison of center of pressure (COP) and ground reaction force (GRF) parameters between young and older adults during the gait task. **(B)** Comparison of COP, GRF, and temporal parameters between young and older adults during the sit-to-stand (STS) task. Data are presented as mean ± standard deviation. ns, not significant; *P < 0.05, **P < 0.01, ****P < 0.0001.

**Table 2:** Comparison of observational indicators between groups (mean ± SD).

Projects	Young group (*n* = 13)	Older group (*n* = 16)	*t* value	*P* value
Gait task				
COP-AP	1.981 ± 0.984	5.725 ± 1.798	6.720	< 0.0001
COP-ML	0.755 ± 0.392	3.177 ± 2.605	3.311	0.0026
COP-TL	2.093 ± 1.008	6.177 ± 1.991	6.715	< 0.0001
GRF-P1	0.995 ± 0.167	0.663 ± 0.172	5.251	< 0.0001
GRF-P2	1.074 ± 0.183	0.750 ± 0.182	4.764	< 0.0001
STS task				
COP-A	0.646 ± 0.106	0.838 ± 0.236	2.700	0.0118
COP-P	0.240 ± 0.238	0.330 ± 0.173	1.171	0.2518
COP-TL	1.128 ± 0.358	1.464 ± 0.830	1.357	0.1856
GRF-Max	0.911 ± 0.425	0.322 ± 0.434	3.668	0.0011
MT	2.605 ± 1.482	2.541 ± 0.973	0.139	0.8909
T-SO	0.323 ± 0.178	0.302 ± 0.132	0.363	0.7196

COP, center of pressure; COP-A, anterior COP displacement; COP-P, posterior COP displacement; COP-AP, anteroposterior COP displacement; COP-ML, mediolateral COP displacement; COP-TL, total COP path length; GRF, ground reaction force; GRF-Max, maximum GRF; GRF-P1, first GRF peak; GRF-P2, second GRF peak; MT, movement time; T-SO, time to seat-off; STS, sit-to-stand.

During STS, older adults showed significantly greater COP-A, indicating altered anterior weight-transfer strategy. GRF-Max was significantly lower in older adults, suggesting reduced lower-limb force generation during postural transition. COP-P, COP-TL, MT, and T-SO did not differ significantly between groups. These results indicate that gait and STS reveal different aspects of age-related motor decline: gait was characterized by increased COP instability and reduced GRF peaks, whereas STS was characterized mainly by altered anterior weight transfer and reduced peak force generation.

## Discussion

This exploratory study provides a multimodal characterization of central and peripheral features of lower-limb motor aging by integrating fMRI-DTI-derived structure–function decoupling with muscle synergy similarity, intermuscular coherence, center-of-pressure dynamics, and ground reaction force during gait and sit-to-stand transitions (Seidler et al., 2010; Rosso et al., 2013; Montero-Odasso et al., 2022; Clark, 2015). Brain SDI differences were regionally concentrated in basal ganglia–striatal, cingulate, and insular systems (Holtzer et al., 2014; Wilson et al., 2019; Grillner and Robertson, 2015; Clark and Taylor, 2011). Notably, age-group-adjusted cross-modal analyses revealed a convergent, although nominal, STS-related pattern: both muscle synergy similarity and mean IMC were positively associated with SDI in the right ventromedial putamen (ROI226) and right dorsal area 23 (ROI176). Because these associations did not survive FDR correction across the candidate ROIs, they should be interpreted as exploratory signals rather than region-specific evidence of brain–muscle coupling. Nevertheless, their convergence across two complementary neuromuscular measures identifies these regions as candidates for future studies examining individual variability in postural-transition control. More broadly, the present findings are consistent with a distributed central–peripheral framework of mobility aging rather than an exclusively musculoskeletal explanation (Seidler et al., 2010; Rosso et al., 2013; Holtzer et al., 2014; Ting et al., 2015).

The basal ganglia-striatal system provides a plausible neurobiological context for the nominal putaminal association observed during STS. The basal ganglia contribute to action selection, movement scaling, automaticity, and the organization of posture and locomotion (Clark, 2015; Clark and Taylor, 2011; Schenkman et al., 1990). Within this system, the putamen is strongly involved in sensorimotor processing and the execution of learned or relatively automatic motor programs (Clark and Taylor, 2011; Schenkman et al., 1990). STS requires rapid transition from a stable seated posture to coordinated forward momentum, lower-limb extension, and final postural stabilization (Bohannon, 2012; Takakusaki, 2017; Schenkman et al., 1990). The nominal association between right ventromedial putamen SDI and both STS synergy similarity and IMC may therefore indicate that individual differences in striatal structure–function organization covary with the organization of peripheral neuromuscular control during this demanding postural transition. Importantly, however, these associations did not survive FDR correction, and the present cross-sectional data cannot determine whether putaminal organization contributes to, compensates for, or simply covaries with peripheral motor differences.

The cingulate finding may provide a complementary interpretation to the striatal result. The right dorsal area 23 is located within the posterior cingulate cortex, a heterogeneous region involved in integrating information relevant to spatial orientation, cognition, and sensorimotor processing. Notably, dorsal area 23 has been reported to show preferential functional relationships with cingulate motor regions, suggesting a potential role in linking sensory and contextual information with motor-related processing (Leech and Sharp, 2014; Vogt et al., 2006). In the present study, the right dorsal area 23 (ROI176) showed an age-group difference in SDI and also exhibited nominal positive associations with both STS muscle synergy similarity and mean IMC after adjustment for age-group membership. This convergence suggests that individual differences in cingulate structure–function organization may covary with peripheral neuromuscular coordination during postural transitions. However, these cross-modal associations did not survive FDR correction across the candidate ROIs and should therefore be interpreted as exploratory rather than as evidence of a specific cingulate-neuromuscular mechanism. Together with the nominal associations observed in the right ventromedial putamen, these findings identify cingulate and striatal regions as candidate neural correlates warranting further investigation in larger, independent cohorts.

At the peripheral level, muscle synergy similarity provides an interpretable measure of how closely an individual’s neuromuscular coordination pattern resembles a young-adult reference organization (Ting et al., 2015; Bizzi and Cheung, 2013; Gogolla, 2017). Older adults showed markedly lower synergy similarity than young adults during both gait and STS, indicating greater divergence from the young-reference spatial organization of muscle recruitment. This finding is consistent with previous reports that aging is accompanied by reorganization of muscle synergies during locomotor and daily-living tasks (Dussault-Picard et al., 2024; Monaco et al., 2010). The STS task showed an additional group difference in the number of extracted modules and broader contributions from trunk, hip, knee, ankle, calf, and intrinsic foot muscles. Given the rapid anterior weight transfer, lower-limb extension, trunk stabilization, and terminal postural control required during STS (Gogolla, 2017; Farina and Negro, 2015), this more distributed recruitment pattern may represent an adaptive strategy for maintaining stability when force-generation capacity and sensorimotor efficiency are reduced. However, because compensation was not directly tested, this interpretation should remain provisional.

IMC provides information complementary to muscle synergy analysis by quantifying frequency-domain synchronization between muscles and the extent of shared neural input (Boonstra, 2013; Halliday et al., 1998; Weersink et al., 2021). During gait, older adults showed lower mean IMC and lower IMC network efficiency, and a distributed set of muscle–muscle connections in Phase 3 survived NBS correction. These findings are consistent with previous reports of age-related modulation of beta-band intermuscular coherence during walking (Dos Santos et al., 2020) and suggest reduced integration of common neuromuscular drive during gait in older adults. In contrast, neither mean IMC nor IMC network efficiency differed significantly between groups during STS. Interestingly, however, individual variation in STS IMC showed nominal age-group-adjusted associations with SDI in ROI226 and ROI176. Thus, group-level differences and cross-modal individual-difference associations were not necessarily parallel: gait showed clearer age-group differences in IMC, whereas STS showed the more apparent exploratory brain-IMC associations. This distinction suggests that task-related central–peripheral relationships may not be captured solely by comparing group means and warrants confirmation in larger datasets.

The COP and GRF results provide complementary biomechanical context for the observed neuromuscular differences (Mehdizadeh, 2018; Manor et al., 2016). During gait, older adults exhibited greater COP excursion together with lower first and second GRF peaks, whereas STS was characterized by greater anterior COP displacement and lower peak GRF. Greater COP excursion in older adults has been associated with altered dynamic postural control, whereas reduced GRF during gait and STS is consistent with reduced loading, propulsion, and lower-limb force-generating capacity (Boonstra, 2013). These biomechanical findings should be viewed as parallel behavioral features rather than direct consequences of the observed SDI, synergy, or IMC alterations, because the present cross-sectional analyses did not test mediation or causal pathways.

A major clinical implication of this work is that age-related mobility decline may require dual targeting of central and peripheral systems (Seidler et al., 2010; Montero-Odasso et al., 2022; Sherrington et al., 2019). On the central side, the basal ganglia–striatal system, particularly the bilateral ventral caudate and right ventromedial putamen, may represent candidate targets for interventions designed to enhance motor automaticity, action selection, and gait/postural control (Clark, 2015; Grillner and Robertson, 2015; Clark and Taylor, 2011). Although conventional non-invasive brain stimulation mainly targets cortical regions, future protocols may consider cortical–subcortical network modulation through motor, premotor, supplementary motor, and cingulate-related circuits connected with the striatum (Sherrington et al., 2019; Manor et al., 2016; Hsu et al., 2015). On the peripheral side, rehabilitation should not only strengthen isolated muscles but also restore coordinated recruitment of hip extensors, knee extensors, ankle plantarflexors/dorsiflexors, intrinsic foot muscles, and trunk stabilizers during gait and STS (Ting et al., 2015; Schenkman et al., 1990; Hanawa et al., 2017; Shen et al., 2017). The integration of SDI, muscle synergy similarity, IMC, COP, and GRF may therefore help identify individualized central and peripheral targets for fall prevention, mobility preservation, and rehabilitation planning (Sherrington et al., 2019).

From a translational perspective, the present findings support further investigation of integrated central and peripheral markers rather than immediate specification of therapeutic or rehabilitation targets. The right ventromedial putamen and right dorsal area 23 may be considered candidate regions for future longitudinal, task-based, and independently replicated studies of postural-transition control, but the current evidence is insufficient to justify direct neuromodulatory targeting or individualized treatment selection. Likewise, the peripheral findings suggest that rehabilitation research may benefit from considering coordinated recruitment across trunk, hip, knee, ankle, and foot muscles in addition to isolated muscle strength (Ting et al., 2015; Farina and Negro, 2015; Boonstra, 2013; Hsu et al., 2015). Future studies should determine whether these candidate central–peripheral relationships are reproducible in independent cohorts and whether longitudinal changes in neuromuscular coordination are accompanied by changes in cortical–subcortical network organization. Existing non-invasive stimulation studies in older adults provide a methodological precedent for investigating network-level modulation, although they do not directly validate the regions identified here (Zhang et al., 2023; Tseng et al., 2024; Wei et al., 2025; Hanawa et al., 2017).

Several limitations should be considered when interpreting these findings. First, the cross-sectional design does not allow causal inference regarding whether altered basal ganglia–striatal SDI drives neuromuscular reorganization or reflects adaptation to peripheral motor decline. Second, the sample size was modest, and replication in larger cohorts is required to confirm the stability of ROI-specific SDI associations and task-specific IMC patterns. Third, resting-state fMRI was acquired separately from the movement tasks; therefore, the observed cross-modal associations reflect relationships between resting-state brain organization and task-related neuromuscular measures rather than simultaneous or task-evoked brain–muscle coupling. Future studies combining longitudinal follow-up, mobile neuroimaging, task-based paradigms, and targeted interventions are needed to determine whether basal ganglia-striatal SDI and peripheral neuromuscular metrics can predict mobility decline or rehabilitation responsiveness.

## Conclusion

Healthy aging was associated with task-dependent differences in lower-limb neuromuscular coordination, biomechanical control, and regional brain structure–function organization. Older adults showed lower muscle synergy similarity during gait and STS, reduced gait-related intermuscular coherence and network efficiency, and altered COP and GRF patterns. Age-group differences in SDI were concentrated in basal ganglia-striatal, cingulate, and insular regions. In exploratory age-group-adjusted analyses, STS muscle synergy similarity and mean IMC showed convergent nominal associations with SDI in the right ventromedial putamen and right dorsal area 23, although these associations did not survive FDR correction. These findings provide multimodal evidence of parallel central and peripheral reorganization during healthy aging and identify candidate brain–neuromuscular relationships for confirmation in larger longitudinal and task-based studies.

## Data Availability

The original contributions presented in the study are included in the article/supplementary material, further inquiries can be directed to the corresponding authors.
